# Anesthetic Management of a Patient With McArdle Disease: A Case Report and Review of the Literature

**DOI:** 10.7759/cureus.40092

**Published:** 2023-06-07

**Authors:** Rohini Kotha, Anastasia Jones, Gregory T Girgenti, Daniel A Nahrwold

**Affiliations:** 1 Anesthesiology, H. Lee Moffitt Cancer Center & Research Institute, Tampa, USA; 2 Anesthesiology, University of Florida College of Medicine, Gainesville, USA

**Keywords:** myoglobinuria, malignant hyperthermia, postoperative fatigue, acute renal failure, rhabdomyolysis, hypoglycemia, general anesthesia, glycogen storage disorder type v, mcardle disease

## Abstract

McArdle disease (glycogen storage disorder type V) is a rare inherited condition resulting in impaired energy metabolism. Challenges in anesthetized patients with McArdle disease include hypoglycemia, rhabdomyolysis, myoglobinuria, acute renal failure, and postoperative fatigue. We review the literature and discuss a successful anesthetic that had no perioperative complications for a patient with McArdle disease undergoing robotic-assisted lung wedge resection. Preoperatively, we obtained a complete blood count, chemistry panel, and creatine kinase level. Intraoperatively, we proceeded with general endotracheal anesthesia and monitored point-of-care electrolytes, hemoglobin, and blood glucose. The patient had an uneventful postoperative recovery and was discharged home on postoperative Day 3. Patients with McArdle disease can undergo safe surgery with appropriate perioperative planning and a well-managed anesthetic. Efforts should focus on mitigating the risks of hypoglycemia, rhabdomyolysis, myoglobinuria, acute renal failure, and postoperative fatigue.

## Introduction

McArdle disease (MAD) is also known as glycogen-storage disorder (GSD) type V and is caused by mutations in the glycogen phosphorylase, muscle-associated (PYGM) gene (discovered in 1984), which encodes glycogen phosphorylase [1]. Glycogen phosphorylase catalyzes the rate-limiting step in glycogenolysis (the breakdown of glycogen into glucose-1-phosphate).

The deficiency or absence of this enzyme results in the inability to release glycogen from the muscle and the accumulation of glycogen in the skeletal muscle, making anaerobic glycolysis impossible. This results in depleted adenosine triphosphate, no rise in lactate, and, subsequently, the destruction of muscle cells during exercise or during diminished blood supply. Symptoms of MAD include painful muscle cramping and weakness during physical exertion [2]. MAD is inherited in an autosomal recessive pattern. The exact prevalence is not known, but the occurrence is rare and estimated to range from 1 in 50,000 to 1 in 200,000 in the United States [3].

Because of the rarity of MAD, specific anesthetic complications are difficult to study. In this report, we review the unique anesthetic-related concerns of a patient with MAD and summarize the current evidence and recommendations in the literature.

## Case presentation

A 72-year-old Jehovah’s Witness patient with a history of MAD, hypertension, hyperlipidemia, gastroesophageal reflux disease, and osteoarthritis presented to Moffitt Cancer Center for robotic-assisted, left-lung lower-lobe wedge resection, possible lobectomy, and possible thoracotomy. He had a 2.3 cm (18)F-fluorodeoxyglucose-avid lung mass abutting the pericardium in the left lower lobe. His surgical history was significant for head and abdominal injuries caused by a farming accident at five years of age, though he could not recall any details about those procedures.

The patient's medications included losartan, colesevelam, pantoprazole, cetirizine, and psyllium. He was a lifelong nonsmoker with no history of alcohol or recreational drug use. He reported limited physical activity caused by a lack of stamina, muscle cramping, and shortness of breath related to asthma and MAD. He would not accept donated blood products but would accept the use of cell-saver blood if a continuous circuit was maintained.

On examination, he had 5/5 strength in the bilateral upper and lower extremities. Pulmonary-function testing showed no obstructive defect based on the forced expiratory volume in one second/forced vital capacity (FEV1/FVC) ratio, normal flow-volume loop, and normal single-breath diffusing capacity for carbon monoxide. Arterial blood gas showed normal oxygenation. Preoperative tests were notable for hemoglobin 15 g/dL, platelet count 282 k/uL, international normalized ratio 1.0, activated partial thromboplastin time 30.9 seconds, creatine 0.6 mg/dL, potassium 4.3 mmol/L, glucose 96 mg/dL, and creatine kinase 851 U/L.

General endotracheal anesthesia was planned for the patient. Two mg of midazolam was given intravenously in the preoperative area. In the operating room, the patient was attached to standard American Society of Anesthesiologists monitors and preoxygenated with 100% FiO_2_ (fraction of inspired oxygen). General anesthesia was induced intravenously with 100 μg fentanyl, 100 mg lidocaine, and 150 mg propofol. Muscle relaxation was achieved with 40 mg intravenous rocuronium. A 37-French left-sided double-lumen endotracheal tube was placed using a Macintosh-4 blade, and its position was confirmed with a fiberoptic bronchoscope. A 20-gauge left radial arterial line was placed, and additional 16-gauge intravenous access was obtained in the right hand.

General anesthesia was maintained with a continuous intravenous propofol infusion and intermittent boluses of intravenous dexmedetomidine, fentanyl, and hydromorphone. A SedLine (Masimo Corporation, Irvine, CA) monitor was placed to monitor the depth of the anesthetic with numerical values between 35 and 45, indicating an adequately anesthetized patient. Intravenous 5% dextrose in lactated Ringer’s solution was infused at 150 mL/hour for the duration of the operation. Point-of-care electrolytes, hemoglobin, and glucose were monitored during the procedure and remained within normal limits.

A wedge resection of the left lower lobe nodule was performed and sent to pathology for a frozen section, which returned positive for spindle-cell neoplasm, likely neuroendocrine. The surgeon then completed a left lower lobectomy. The patient tolerated the procedure and single-lung ventilation well. There were no intraoperative complications.

The surgical duration was three hours, and the estimated blood loss was 40 mL. Reversal of muscle relaxation was achieved with intravenous sugammadex after the patient had 2/4 twitches based on peripheral nerve stimulation. The patient emerged from general anesthesia, and his trachea was safely extubated. He remained stable in the postanesthesia care unit. The patient was admitted to a regular ward bed, had an uneventful postoperative recovery, and was discharged home on postoperative Day 3.

## Discussion

**Figure 1:**
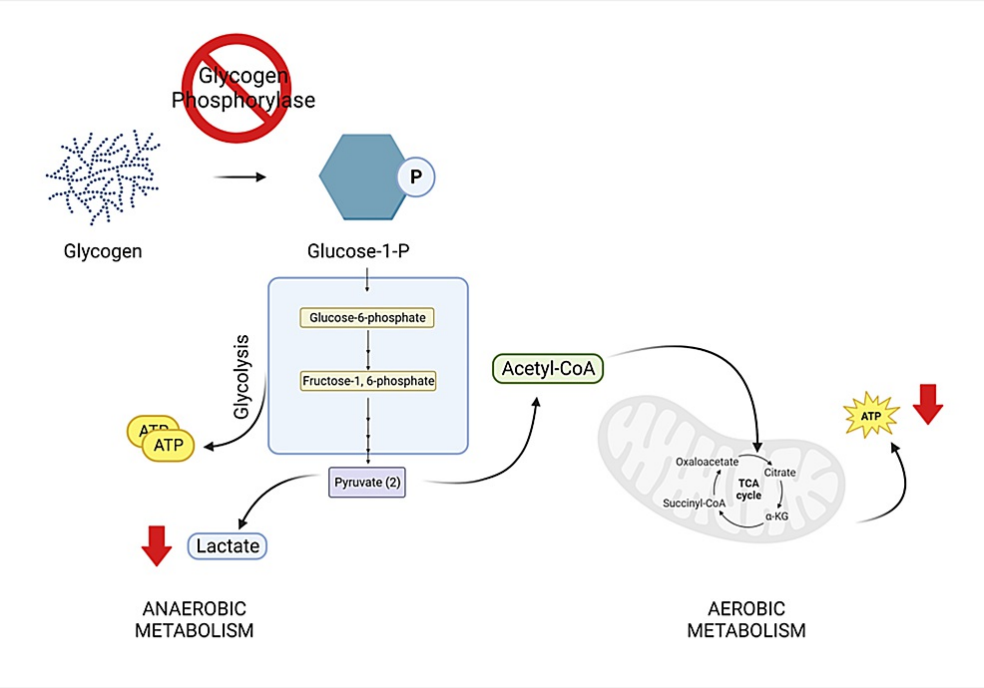
Pathophysiology of McArdle disease Inactivated glycogen phosphorylase enzyme results in the inability to release glucose-1-P from intracellular glycogen stores to participate in glycolysis, followed by anaerobic and aerobic metabolism. Consequently, lactate does not rise and adenosine triphosphate production decreases in the muscle cells during exercise. Abbreviations: α-KG, alpha-ketoglutarate; ATP, adenosine triphosphate; CoA, coenzyme A; P, phosphate; TCA, tricarboxylic acid

The pathophysiology of MAD arises from an inability to release glucose from intracellular glycogen storage, leading to depleted adenosine triphosphate and no rise in lactate in the muscle cells during exertion, which subsequently results in rhabdomyolysis (Figure 1). Muscle pain, muscle cramps, stiffness, and early fatigue on exertion that improves with rest are characteristics of this disorder. Our patient did indeed have poor exercise tolerance and muscle cramping. Some patients, however, experience the “second-wind” phenomenon associated with the activation of fatty acid oxidation in which heart rate suddenly decreases and an improved exercise tolerance occurs after a few minutes of exercise. Our patient also complained of dyspnea on exertion, which may have been associated with asthma rather than MAD. Shortness of breath is not usually associated with MAD.

The unique anesthetic considerations for patients with MAD stem from rhabdomyolysis and include potential complications such as hypoglycemia, myoglobinuria, acute renal failure, and postoperative fatigue [2]. Because of the rarity of this disorder, the current understanding of MAD and anesthetic interactions is based on a series of case reports, retrospective clinical reviews, and expert opinions. A review of the current literature (published in English) on MAD and anesthesia demonstrated zero research studies and nine case reports comprising 14 patients with 42 anesthetics (Table 1). Overall, these published case reports demonstrate safe anesthetic administration. Reported complications include tachycardia and hypotonia in one case of an otherwise healthy 21-year-old man undergoing general anesthesia for otoplasty [2]. After an extended evaluation for elevated transaminases, the patient was found to have a creatine kinase level >5000 U/L. This led to a referral to a neurologist, a muscle biopsy, and exercise testing demonstrating no rise in lactate, all confirming the diagnosis of MAD. Interestingly, the same patient underwent three prior general anesthetics without any difficulties.

**Table 1:** Overview of case reports describing anesthesia in patients with McArdle disease Abbreviations: CPB, cardiopulmonary bypass; E, epidural; F, female; GA, general anesthesia; IVCT, in vitro contracture test; LA, local anesthesia; M, male; MAD, McArdle disease; MH, malignant hyperthermia; MHN, malignant hyperthermia negative; MHS, malignant hyperthermia susceptible; NMB, neuromuscular blockers; POD, post-op day; PONV, postoperative nausea/vomiting; RA, regional anesthesia

Year and citation	Patient (age, gender)	Type of surgery	Type of anesthesia		Anesthetic agents and NMB		Complications			Comments
1984 Coleman P [4]	25 y, F	Urgent cesarian section	GA		Methohexitone, alcuronium, morphine, nitrous oxide		None			Patient insisted on general anesthesia
1986 Rajah A, Bell CF [5]	4 y, M	Adenoidectomy	GA		Tiopentanone, fentanyl, atracurium, nitrous oxide		None			Probable MAD
1988 Samuels TA, Coleman P [6]	31 y, F	Cesarean section	E		LA (bupivacaine)		None			
1989 Isaacs et al [7]	6 y, M	Muscle biopsy	GA		Ketamine		None			Family history of MH; IVCT; MHS
1990 Tzabar Y, Ross DG [8]	64 y, M	Hemicolectomy	GA		Propofol, alfentanil, vecuronium, isoflurane, nitrous oxide		None			
1999 Lobato et al [9]	2 y, M	Repair of tetralogy of Fallot	GA + CPB		Fentanyl, pancuronium, halothane		Hyperthermia, rhabdomyolysis, pulmonary edema, death POD 10			Rhabdomyolysis post bypass, possible adverse reaction to protamine
2005 Bollig et al [10]	72 y, M	Cholesteatoma right ear at 65 y	GA		Fentanyl, propofol		None			
		Glaucoma right eye at 66 y	LA		LA		None			
		Glaucoma left eye at 68 y	LA		LA		None			
	46 y, F	Tonsillecotomy at 5 y	GA		Unknown		None			
		Uterine leiomyoma at 32 y	GA		Fentanyl, thiopentone, dehydrobenzpyridol, enflurane, succinylcholine, pancuronium		None			PONV, headache
	53 y, F	Cleft lip at 8 wk old	GA		Unknown		None			
		Appendectomy at 4 y	GA		Unknown		None			
		Tonsillectomy at 13 y	GA		Unknown		None			
		Muscle biopsy arm at 27 y	GA		Unknown		None			
		Muscle biopsy of shoulder at 28 y	GA		Unknown		None			
		Muscle biopsy at 28 y	LA			LA		None		
		Curettage	Spinal			LA		None		
	40 y, F	Laparoscopy at 34 y	GA			Unknown		None		
		Muscle biopsy at 36 y	LA			LA		None		
	34 y, F	Adenoidectomy at 7 y	GA			Unknown		None		
		Muscle biopsy at 22 y	LA			LA		None		IVCT: MHS
		Tooth extraction	GA			Nontriggering anesthetic		None		
	37 y, F	Muscle biopsy at 24 y	LA			LA		None		IVCT: MHN
		Laparoscopy at 35 y	GA			Nontriggering anesthetic		None		
	52 y, F	Polypectomy at 10 y	LA			LA		None		
		Cholecystectomy	GA			Unknown		None		
		Septoplasty at 39 y	GA			Unknown		None		
		Scar revision at 40 y	GA			Unknown		None		
		Scar revision at 41 y	GA			Unknown		None		
		Curettage at 41 y	GA			Unknown		None		
		Scar revision at 42 y	GA			Unknown		None		
		Scar revision at 43 y	GA			Unknown		None		
		Muscle biopsy at 47 y	LA			Unknown		None		
2013 Bollig G [2]	21 y, M	Otoplasty at 13 y		GA		Unknown			None	PONV
		Tonsillectomy at 19 y		GA		Alfentanil, brevimytal, nitrous oxide			None	
		Septoplasty		GA		Thiopentone, fentanyl, succinylcholine, alfentanil, isoflurane, halothane			Tachycardia and hypotonia	
		Muscle biopsy arm		LA		Local anesthetic			None	
		Osteochondroma resection and bone graft		Spinal + RA		Local anesthetic			None	Use of tourniquet (upper arm) without problems
		Muscle biopsy leg		RA		Local anesthetic			None	IVCT result: malignant hyperthermia susceptible (MHS)
		Septoplasty at 38 y		LA		Local anesthetic			None	Patients wish to use LA instead of general anesthesia as recommended by the surgeon
2011 Chalaeva A [11]	48 y, F	Exploratory laparotomy, rectal tumor excision		GA		Midazolam, fentanyl, propofol, cisatracurium, meperidine			None	

In another case of a two-year-old male undergoing repair of tetralogy of Fallot, the authors reported hyperthermia, rhabdomyolysis, and pulmonary edema coming off of cardiopulmonary bypass and following protamine administration [9]. MH was unlikely because there was no muscle rigidity, no end-tidal CO_2_ elevation, and no response to dantrolene. The authors surmised that severe rhabdomyolysis was caused by protamine and cardiopulmonary bypass in the context of MAD, which was later diagnosed. Unfortunately, this patient developed progressive renal failure and severe sepsis and died on postoperative Day 10. The diagnosis of MAD was established by a skeletal muscle biopsy on postoperative Day 5, showing the absence of skeletal myophosphorylase with accumulated tissue glycogen.

There is no apparent connection to MH within the glycogen storage myopathies, including MAD [12]. There are no case reports of MH and inhalational anesthetic-induced hypermetabolism attributable to MAD. In one review of case reports involving MAD patients having anesthesia by Bollig et al., two patients tested positive on the in vitro contracture test (IVCT) [10]. However, these IVCTs attempting to reveal MH susceptibility in patients with MAD ignore two important points: 1. Contracture testing protocols were validated in patients with a clinical history suspicious for MH, and 2. Contracture tests have not been validated for patients with metabolic myopathies, including MAD. Most likely, positive IVCTs in patients with MAD are the result of a muscle running out of fuel [2]. Admittedly, we chose to proceed with total intravenous anesthesia and avoidance of succinylcholine for our patient given that some case reports in the literature suggest an association between potent inhalational anesthetics, succinylcholine, underlying myopathies, and resultant rhabdomyolysis. This has been most notably described for congenital myopathies, such as Duchenne and Becker muscular dystrophies, with reports of unpredictably worsened rhabdomyolysis complicated by hyperkalemic cardiac arrest with potent inhalational anesthetics even without the administration of succinylcholine [13,14].

**Figure 2:**
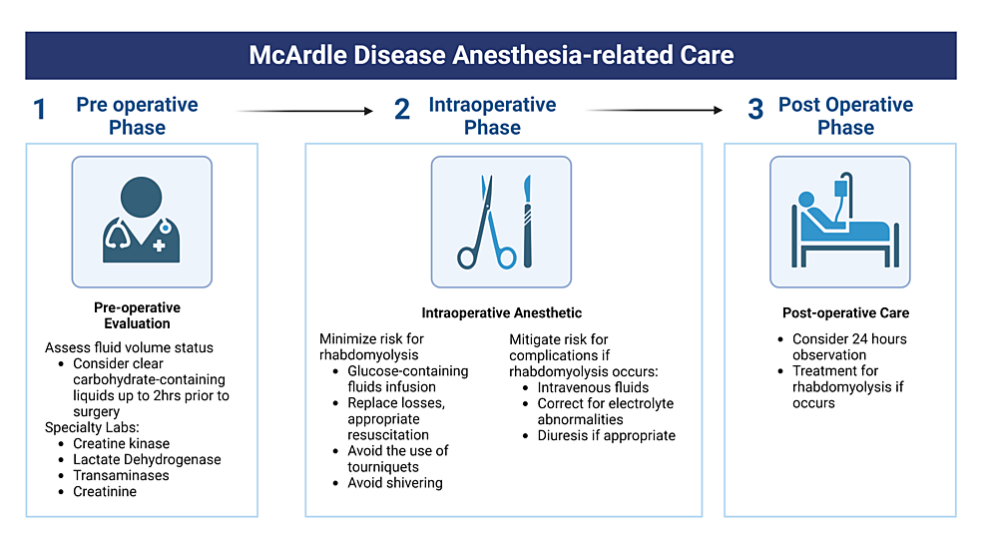
Summary of anesthetic considerations for patients with McArdle disease

A summary of anesthetic considerations is outlined in Figure 2. Preoperative evaluation should include baseline creatine kinase because values can be increased from five-fold to 18-fold even at rest [15]. Lactate dehydrogenase, transaminases, and creatinine should also be included in preoperative laboratory testing to establish baseline values. Additionally, patients should be adequately hydrated and encouraged to drink glucose-containing clear liquids for up to 2 hours preoperatively.

The intraoperative anesthetic should include monitoring blood pressure, heart rate, pulse oximetry, end-tidal CO_2_, and core temperature. Glucose infusion may improve the availability of energy substrates, although it has not been shown to prevent rhabdomyolysis. For example, a case report by McMillan et al. describes a 23-year-old woman with rhabdomyolysis developing after 149 minutes of pushing and vaginally delivering a healthy baby [16]. In this case, creatine kinase peaked at 28 500 U/L on postpartum Day 2 and eventually decreased to 9708 U/L by postpartum Day 5, despite a peripartum intravenous infusion of lactated Ringer’s solution with 5% dextrose at 150 mL/hour to maintain blood glucose >5.6 mmol/L and increased to 200 mL/hr once rhabdomyolysis was discovered, approximately 45 minutes after delivery. Thus, muscular damage is best prevented by avoiding significant exertion, treating shivering, and avoiding tourniquets, though a tourniquet was well-tolerated in a case report by Bollig [2].

Postoperative care can usually follow the normal protocol for patients without MAD. However, in case rhabdomyolysis ensues, consider observational care for 24 hours with follow-up creatine kinase, lactate dehydrogenase, and creatinine laboratory testing.

## Conclusions

MAD presents unique perioperative challenges to the anesthesiologist, including hypoglycemia, rhabdomyolysis, myoglobinuria, acute renal failure, and postoperative fatigue. There is no evidence of clinical MH episodes induced by the exposure of MAD patients to potent inhalational anesthetics. An association between potent inhalational anesthetics and rhabdomyolysis has never been reported in patients with MAD. Similarly to other inherited myopathies, patients with MAD should not receive succinylcholine due to the potential to trigger acute rhabdomyolysis, hyperkalemia, and cardiac arrest. By executing a well-planned anesthetic, patients with MAD can undergo safer surgery with minimal perioperative complications.
